# Under pressure: the interaction between high-stakes contexts and individual differences in decision-making in humans and non-human species

**DOI:** 10.1007/s10071-023-01768-z

**Published:** 2023-03-29

**Authors:** Meghan J. Sosnowski, Sarah F. Brosnan

**Affiliations:** 1 Department of Psychology, Georgia State University, P.O. Box 5010, Atlanta, GA 30302-5010 USA; 2 Language Research Center, Georgia State University, Atlanta, GA USA; 3 Center for Behavioral Neuroscience, Georgia State University, Atlanta, GA USA; 4 Neuroscience Institute, Georgia State University, Atlanta, GA USA

**Keywords:** Pressure, “Choking”, Non-human species, Stress response, Hormones

## Abstract

Observed behavior can be the result of complex cognitive processes that are influenced by environmental factors, physiological process, and situational features. Pressure, a feature of a situation in which an individual’s outcome is impacted by his or her own ability to perform, has been traditionally treated as a human-specific phenomenon and only recently have pressure-related deficits been considered in relation to other species. However, there are strong similarities in biological and cognitive systems among mammals (and beyond), and high-pressure situations are at least theoretically common in the wild. We hypothesize that other species are sensitive to pressure and that we can learn about the evolutionary trajectory of pressure responses by manipulating pressure experimentally in these other species. Recent literature indicates that, as in humans, pressure influences responses in non-human primates, with either deficits in ability to perform (“choking”) or an ability to thrive when the stakes are high. Here, we synthesize the work to date on performance under pressure in humans and how hormones might be related to individual differences in responses. Then, we discuss why we would expect to see similar effects of pressure in non-humans and highlight the existing evidence for how other species respond. We argue that evidence suggests that other species respond to high-pressure contexts in similar ways as humans, and that responses to pressure are a critical missing piece of our understanding of cognition in human and non-human animals. Understanding pressure’s effects could provide insight into individual variation in decision-making in comparative cognition and the evolution of human decision-making.

## Pressure and how it impacts performance

Each day, humans and other animals face situations in which beyond ability, making the appropriate choice or performing well has bearing on the benefits or consequences of their action. Pressure—or a situation in which an individual’s outcome relies on their own performance as well as their ability—is a key factor in many domains of human performance, from sports to academics. Such pressure is usually associated with a threat to that individual’s physical or psychological well-being. This is true in the life-or-death situations, but even in situations in which threats are purely psychological (for instance, not embarrassing oneself when speaking publicly, or avoiding the potential loss of a financial opportunity), pressure can impact an individual’s ability to perform well. Indeed, in humans, we often see evidence of a biological stress response to the experience of pressure, in the absence of an actual physical threat that would typically induce that stress response. By our definition, pressure is present in almost all situations in which an animal needs to complete an action or task, albeit often at a low level that may not impact responses. Therefore, it may be useful to think of pressure level of a situation as a spectrum rather than a binary feature. Increased pressure as the result of high stakes can induce striking deficits in cognitive performance, a phenomenon that has been colloquially referred to as “choking” under pressure. However, there are individual differences—rather than choking, some people—or those in specific circumstances—show a boost in performance, or “thrive” under pressure. This may be due to an increase in motivation and suggests that there is interplay between motivational systems and stress systems that result in variable performance outcomes. Understanding why pressure sometimes helps us and why cognition sometimes fails us when performing has been a key focus of research into performance under pressure.

Choking under pressure has been, to date, considered a human phenomenon, but humans are not unique in facing high-pressure situations. Non-human animals also experience high-stakes, life-or-death situations in which making the correct decision can result in survival while acting inappropriately can quite literally have fatal consequences. Additionally, many animals have cognitive systems like those that are susceptible to interference from pressure in humans, suggesting that there is reason to predict an effect of pressure in situations and tasks that engage these systems. Despite this, the possibility that non-humans might experience pressure when engaging in cognitive tasks and the impact of deficits as a result of pressure has been largely ignored as a subject of specific study. This oversight is curious, as understanding if non-humans react to pressure and whether there is individual variation in likelihood of choking would allow us to better understand the role that coping with pressure might have played in our own evolution. Further, such understanding could have crucial explanatory value in our interpretation of within-species variability in comparative cognition. Thus, in this review, we argue that responses to pressure are a critical missing piece of our understanding of cognition in both humans and other animals.

## Performance and cognition under pressure in humans

The vast majority of research on the influence of pressure on performance and choking under pressure has been in samples of human subjects and has focused on those who suffer performance deficits under pressure. Much of the research has focused on understanding who chokes (Sattizahn et al. 2016), when they choke (Beilock and Gray 2007), and in what contexts they choke (DeCaro et al. 2011). Responses to pressure and the ability to perform under that pressure varies widely among individual people, but in predictable ways, at least among the samples that have been studied. First, experts who are performing a procedural task at which they are highly experienced are far more likely than novices to choke when asked to complete that task under unusually high pressure (or under the *perception* of unusually high pressure). For instance, expert golfers were more likely to miss a putt than novices when they were induced to think about the stakes of the putt (Beilock and Carr 2001). Professional soccer players showed a similar effect of expertise when asked to complete an exercise in which they needed to accurately dribble a ball (Jackson et al. 2006), suggesting that individuals who have reached the point where they have “automatized” a given task are more susceptible to a deficit in performance.

Although there are a reasonable number of studies on the effect of pressure on performance in humans, it is important to note that most of this previous research has been focused on only a subset of samples, which are largely Western, Educated, Industrialized, Rich, and Democratic (WEIRD; Henrich et al. 2010). Such focus on one evolutionarily anomalous subset of the human population, while unfortunately not uncommon in cognitive research (Rad et al. 2018), limits our ability to fully understand how culture, life circumstance, and experience impact stress responses and pressure effects. Further, the assumption of a universal human trait or response ignores the likelihood that there are within-culture and between-culture variation of response, and that these sources of variation likely interact to produce responses (Kline et al. 2018). The study of pressure is no different in this regard—the overwhelming majority of research on performance under pressure has been conducted in WEIRD human samples with no comparison to other cultural groups. One exception to this homogeneity in sampling, in which in which Ariely and colleagues studied how larger monetary incentives led to lower overall earnings playing a series of games (that is, worse performance) in both an Indian sample and a WEIRD undergraduate sample, found no evidence of a difference in negative response to high-incentive pressure between the two samples (although a direct comparison between the two groups with identical tasks is not made; 2009). A more recent study (Aravind et al. 2022) into pre-competition anxiety in Indian athletes across five sports suggested that emotional intelligence had a negative relationship with competition anxiety similar to that observed in the Eurocentric anxiety literature, but that the magnitude of this relationship was much smaller than those previously reported. However, this 2022 pilot study did not study the impact of pressure to perform on outcomes specifically, so these results are not directly comparable to the existing choking literature from WEIRD samples. Unfortunately, to date there has been no other research on cultural groups beyond WEIRD samples and Indian samples, making it difficult to know whether these patterns generalize to other populations and what other effects are yet undiscovered.

Despite this limitation in our ability to generalize across all humans, there are some predictable patterns in the studies from WEIRD samples that have been conducted. For instance, focusing a person’s attention on their actions during a task seems to increase the likelihood of failure under pressure. In a task where participants had to skillfully manipulate a ball through an apparatus under pressure, the instruction to be acutely aware of what their hands were doing as they completed the task increased the likelihood of participants failing under the pressure (Baumeister and Showers 1986). Post-task, self-reports of self-consciousness were also associated with higher likelihood of failing (Baumeister and Showers 1986). Similarly, basketball players who reported self-consciousness along with trait anxiety were more likely to miss free throws when put into high-pressure conditions (Wang et al. 2004). Therefore, adding conscious attention to a practiced procedure seems to change the way that human participants are completing the task. In an interesting and promising intervention approach, targeted self-consciousness training seems to improve performance under pressure. Soccer players that practiced being aware of their own performance during penalty shots performed better in true high-pressure situations (Reeves et al. 2007).

Such performance deficits are not limited to physical performance or procedural memory, but can also affect performance on cognitive tests. Math performance has been the most studied type of performance within this domain, and has implicated working memory as a cognitive system that is particularly impacted by pressure. People tasked with solving complex math problems were more likely to choke under high pressure when they had high-working-memory scores under typical pressure conditions (Mattarella-Micke et al. 2011). One proposed explanation for this is that people high on the spectrum of working memory capacity might be able to use their superior capacity to rely on more cognitively effortful strategies to complete the tasks under normal pressure. However, under high pressure, their ability to use such cognitively taxing strategies is reduced when working memory is strained by the pressure load (Beilock and DeCaro 2007; Beilock 2008). If this is the case, then we might be especially likely to see negative effects of pressure when a cognitive task relies on working memory in some way.

Other types of working memory tasks (and tasks that involve fluid intelligence, a process related to working memory) beyond mathematical problem-solving are also affected by pressure. For instance, the Raven’s Standard Progressive Matrices (SPM) task involves visual completion problems that require participants to select the final image to complete an array. Notably, the matrices vary in working memory demands, which could make some trials in the task particularly prone to deficits under pressure. Indeed, participants were influenced negatively by pressure only on high-working-memory demand trials. High-working memory capacity participants also showed a greater accuracy deficit under high pressure (Gimmig et al. 2006), supporting that working memory processes are susceptible to pressure in areas beyond mathematical reasoning. As another example, the Simon task (Simon 1990) requires participants to ignore irrelevant location cues to respond correctly to a visual color cue by pressing one of two buttons, a procedure that involves controlling processing in working memory by directing attention. When participants were tasked with completing trials in the Simon task while in the presence of an experimenter (a situation that might increase pressure through monitoring), they were significantly more likely to show an effect of the interference of the irrelevant cues (Belletier et al. 2015). This effect was heightened in high working memory capacity participants (Belletier et al. 2015). Working memory (and processes that are related to it, such as executive attention or more general fluid intelligence) has been admittedly the most well-studied process in terms of purely cognitive performance, but it also has been particularly well-supported by those studies. 

Although the aforementioned deficits of homogenous sampling limit our ability to generalize, the human studies from WEIRD populations have been used in developing theories to explain how pressure impacts cognition and working memory specifically to produce a deficit in performance (Yu 2015). All of these theories relate in some way to the attentional demands that acute stress places on a performing individual and how that shifted attention negatively affects the ability to complete a task or make a decision. However, the theories differ in exactly what causes this attentional shift and to where that attention is reallocated. The distraction account suggests that the experience of pressure is an uncomfortable one, and that this discomfort is distracting enough to a person that their performance in a task suffers (Wine 1971). In essence, this hypothesis posits that performance deficits are due to attention being directed away from the task that needs to be completed. When under pressure, a participant’s attention is largely focused on the uncomfortable experience of the acute stress, and this distraction may lead to slower responses or slower cognitive processing in task-relevant regions. This reallocation of attention takes resources away from the cognitive processing required to complete the task, resulting in a deficit in available attentional resources to complete the task, which causes an observed decrease in performance when under pressure. Additionally, emotional or affective regulation in the face of stressful situations may add to the cognitive effort needed to complete a task under pressure.

In contrast, the explicit monitoring account proposes that pressure causes a metacognitive shift from an automatic process to one in which the person is acutely aware of their actions, such that attention is turned toward the task that needs completing, but in a way that heightens self-consciousness. The explicit monitoring account suggests that this attentional shift to monitoring task performance while performing said task counterintuitively impedes ability to perform. This is consistent with the fact that many of the activities in which experts “choke” are tasks that are rooted in procedural memory (for instance, the previously discussed research involving expert golfers or soccer players). Additionally, this effect does not seem to be limited to procedural memory, as there is evidence across several domains that self-consciousness while performing a task drastically reduces performance on that task even when the task involves mathematical reasoning (Wine 1971; Lyons and Beilock 2012a) or performing in front of a supportive audience (Wallace et al. 2005).

Finally, the over-arousal account (sometimes called the over-motivational account) hypothesizes that the pressure to perform is linked to a desirable incentive to complete a task, and that this incentive heightens the overall arousal of the individual to the point that the brain cannot focus on the task appropriately. The over-arousal account has its basis in the Yerkes-Dodson law (Yerkes and Dodson 1908; Yu 2015), which states that for every task there is an optimal level of arousal that facilitates performing that task, and beyond that threshold additional arousal impedes performance. Like the previously described distraction model, this account is heavily focused on the experience of pressure. However, a key distinction is that in the over-arousal account, there is less of a conscious focus on the uncomfortable sensation but instead a heightened urge to respond quickly, which can lead to errors in performing the task. In accordance with this, the over-arousal account posits that the high incentive to perform increases arousal via motivation, which for simple, procedural tasks improves performance, but for complex or unpracticed tasks might lead to an “overload” in desire that actually impedes effective cognition, as seen in audience effects in social cognitive studies (Zajonc 1965). This difference in response as a result of task complexity is also supported by tasks in which the cardiovascular response was elevated to threat-level heart rate when completing complex tasks in front of an audience (Blascovich and Tomaka 1996). Additionally, an account highly focused on motivation would suggest that by reframing the motivation to be an opportunity of gain, rather than a threat of loss, we might see different results. However, as opportunity framing has not been directly tested as an intervention for those likely to choke, and in fact may be more likely to induce choking (Dunne et al. 2019), further work needs to explore the link between motivation and poor performance. Beyond this evidence, the mechanism by which the over-arousal effect would impact performance under pressure is not well-explained (Yu 2015), which provides an opportunity to explore this interaction between motivation and overarousal further by including physiological signs of arousal in an exploration of pressure.

The three explanations for pressure-induced performance failure are difficult to distinguish in the humans sampled to date, presumably in part because the evidence is not conclusive and in part because they are not mutually exclusive (Table 1). Considering the former, neuroimaging techniques have resulted in some support for all three (Yu 2015). An fMRI study provided evidence that deficits in working memory task performance were related to reduced activity in regions associated with attentional executive function as well as working memory (Qin et al. 2009). This supports a distraction account of “choking,” which predicts that acute stress and high pressure result in the inability to effectively allocate attention to the task. A subsequent neuroimaging study found that an increase in connectivity between executive control and motor cortex regions prior to physical movement was negatively related to performance in a computerized task, suggesting that executive control resources that should have been allocated to completing the tasks were instead allocated to anticipation of the physical performance (Lee and Grafton 2015). However, such an explanation is also not necessarily inconsistent with an overarousal account. People under pressure might anticipate the distracting experience of acute stress combined with the increased arousal of high stakes. Indeed, other research shows that increased cognitive control and management of negative emotions prior to beginning a high-pressure task reduces performance deficits (Lyons and Beilock 2012a), also suggesting arousal is involved. Overall, the data to date suggest a multiple system model or a context-based account (Yu 2015), but it is not clear whether this multiple model system is needed to explain choking, or if such an explanation is simply a result of the fact that we cannot disentangle these possibilities in human subjects. Complicating things further, even once we are able to disentangle these explanations, we still have work to do in understanding what contextual factors are most relevant to each explanation and which individual features would make choking more likely under each explanation. Indeed, not everyone does end up choking, and some individuals even thrive under pressure. The next step, then, is to understand how individual differences may play an important role in how these systems interact to produce choking behavior when under pressure.

**Table 1:** Overview of the different accounts of choking under pressure in existing human literature, with a few selected empirical studies that are consistent with the accounts. Note that the accounts are not mutually exclusive, and some results are consistent with more than one account

Account	Introduced in…	Attention reallocated to…	Sample empirical results consistent with account
Distraction	Wine (1971)	Uncomfortable sensations of the stress response	Qin et al. (2009); Lyons and Beilock (2012a); Mattarella-Micke et al. (2011)
Explicit Monitoring	Baumeister (1984)	Performer’s own actions and avoidance of error	Wallace (2005); Lyons and Beilock (2012a)
Over-arousal/over-motivational	Yerkes and Dodson (1908) (basis in)	High motivation to avoid loss and urge to respond quickly to a task	Ariely et al. (2009), Lee and Grafton (2015), Lyons and Beilock (2012a);, b)

## Individual differences, the stress response, and hormones

One area in which individuals differ is in their response to stress. Pressure likely acts as an added stressor when trying to perform, so it seems logical that the stress response is involved in producing poor performance on cognitive tasks. Further, individual differences in how each individual responds to this stress in-the moment might be related to observed differences in performance. Individual responses to stress can differ both in their overall levels of stress as a result of their environment and life history and in how they cope with immediate, temporally transient stressors in the moment. Importantly, overall stress level and immediate reaction to in-the-moment stress often interact to produce observed behaviors and reactions. Therefore, when considering how individuals react to pressure, and which hormones related to stress might influence those reactions, it is important to understand both the distinction and the potential connection between the effects of chronic and acute stress.

Chronic stress refers to a state of long-term stress over months or years, whereas acute stress is the result of a single threatening situation and occurs in-the-moment at the appearance of a threat. By this definition, pressure most likely represents an acute stressor, in which the situational stakes pose a threat to psychological well-being (and in the case of life-or-death decision-making, to physical well-being). Chronic stress has been well-studied in both humans and other animals, with the overall conclusion that chronic stress usually has negative impacts on body condition, immune response, and cognitive functioning (Sapolsky 1990). In addition, chronic stress impacts the immediate stress response. Previous work found a negative association between increased chronic stress and cortisol reactivity in-the-moment, suggesting that high levels of chronic stress downregulate the impact of any one stressor (Rich and Romero 2005), although this does not necessarily translate into behavioral differences. Therefore, long-term and immediate stress states probably interact to produce any given behavior or decision, and we should be concerned with both chronic and acute stressors when assessing an individual’s behavioral response to a threat and the underlying decision-making processes.

The overall stress response is relatively well-conserved, but the stressors themselves have not remained constant in the evolutionary history of species, nor have the potential outcomes of those stressors. High-pressure situations faced by some human populations today do not always come with life-or-death consequences (Monroe 2008). Instead, they are often lower-stakes but longer lasting and, rather than threatening physical well-being, as something like a predator does, threaten psychological or social well-being and may have many more potential outcomes than simply “fight” or “flee”. Because this shift is evolutionarily recent, however, we still respond as though all threats are physical ones. Thus, what was evolutionarily a beneficial tradeoff among the physical and cognitive systems impacted by the stress response might now represent a misalignment of priorities, since the stress response tends to prioritize physical readiness while neglecting complex cognition. Negative performance effects are likely due to this tradeoff in priorities (de Kloet et al. 1999; Nesse and Young 2000).

Stress responses have been relatively well-conserved among species, particularly in mammals (Selye 1950; Reeder and Kramer 2005), making the role of pressure in performance a good candidate for the comparative approach. When a stressor appears, an individual needs to perceive and interpret it as a threat to its well-being, quickly decide how to react to it, and make the right decision (i.e., the decision that maximizes opportunities for survival and reproduction, not necessarily in that order). Each of these steps requires input from a different part of the body or brain. For instance, specific areas of the brain are responsible for perceiving a threat and making a decision, and those regions then need to communicate with other areas of the body to prepare the individual to physically deal with the threat. The hypothalamic—pituitary—adrenal (HPA) axis connects the brain systems needed to perceive a threat with the somatic systems needed to physically respond to that threat (Selye 1950). Hormones connect many of the steps of this response, making them a key physiological marker of HPA activity (Stratakis and Chrousos 1995). Because hormones are sensitive to context and fluctuate in response to environmental stimuli, they change the likelihood of a specific behavior occurring based on those environmental cues. Since pressure is a response to an environmental cue that likely induces a stress response, it seems likely that hormones might also be altering how animals think, make decisions, and perform as animals engage in cognitive processes under pressure. Therefore, the hormones involved in the hypothalamic–pituitary–adrenal (HPA) axis cascade are obvious potential mechanisms for how pressure might be related to cognitive deficits (although they likely interact with other stress-response mechanisms, which we will discuss later), and using them as markers of the stress response, we may be able to detect how the stress response relates to likelihood of choking.

One key hormone in the HPA response is cortisol, a metabolic steroid hormone that has been widely studied in the context of stress in both human subjects and other animals (Selye 1950; Sapolsky 1988; Sapolsky et al. 2000). Beyond its physical impacts, cortisol also has been shown to impact cognition as part of a feedback loop in the HPA axis, in which cortisol is active not only in the hypothalamus, but also in the hippocampus, which moderates the HPA’s response to stress (McEwen and Sapolsky 1995). The hippocampus is also critical in memory formation, and the presence of cortisol receptors in this region suggests that cortisol could be impacting processes involving hippocampal-based memory. There are also glucocorticoid receptors in the amygdala, a region associated with affect and emotional response. As many accounts of cognitive deficits resulting from pressure involve an affective response, it is reasonable to expect that cortisol might also act in the amygdala to further impact cognitive performance by providing an affective experience that is distracting and unpleasant.

Further, we already have reason to predict that cortisol may be related to the attentional shift predicted in the theories for choking, at least in humans. Increased salivary cortisol in response to psychosocial pressure during a math task has been associated with significant deactivation in the hippocampus (Pruessner et al. 2008). The authors proposed that the hippocampus, a region critical for learning and memory, also has a “default state” of inhibiting the HPA axis and managing individuals’ emotional response or affective state, and that when put under pressure, the hippocampus is unable to both manage the stress response and perform the task at hand, leading to decreased performance on the task (Pruessner et al. 2008). There is also some evidence that cortisol reactivity correlates with performance under pressure, although the one study to date found a sex difference (van den Bos et al. 2009), suggesting that there may be significant individual differences that have not yet been uncovered that impact the relationship between cortisol and pressured performance. As an example, one study found an interaction between working memory capacity (WMC) and salivary cortisol impacting the likelihood of choking on mathematical problems, such that high individuals scoring higher on a WMC scale were more likely to fail under high pressure when they had higher levels of cortisol (Mattarella-Micke et al. 2011). The authors hypothesized that pressure-induced increases in cortisol might add some type of attentional load on working memory. Because high-WMC-scoring individuals might use more complex problem-solving strategies under normal conditions, the additional load of pressure proves too much for even these highly performing individuals if they continue trying to use these strategies when under high pressure (Mattarella-Micke et al. 2011). Importantly, these results are consistent with both the distraction and overarousal explanations of choking in that additional attentional load on working memory would both take resources away from the task at hand (distraction) and would potentially increase arousal beyond the optimal threshold for a complex task like mathematics (overarousal).

Although cortisol seems to be a key component of how an individual responds under pressure, other hormones probably impact performance as well, or interact with cortisol to do so. Although the HPA response occurs within minutes of the stressor’s onset, most of its activity still occurs after the response of the sympathetic nervous system (the sympatho-adrenal response), which is connected by its own, separate set of hormonal messengers, including norepinephrine, epinephrine, and the neurotransmitter acetylcholine. We already know that epinephrine mediates memory performance (this was first reported in rats by Gold and Van Buskirk 1975), but the effects are both dose-dependent and time-dependent (Gold 1987). Because the sympatho-adrenal response to a stressor typically occurs either simultaneously with or immediately prior to the HPA response, it is likely that observable evidence of choking is the result of the hormones in both cascades acting on receptors in the parts of the brain that influence memory, such as the aforementioned hippocampal regions and the amygdala. Indeed, activation of the sympathetic nervous system seems to be a necessary component of cortisol’s negative impact on working memory performance (Elzinga and Roelofs 2005).

Gonadal hormones may also interact with the stress response to produce behavioral responses to pressure, especially since we have evidence both that they impact cognition on their own as well as having an interaction with cortisol. For instance, there has been extensive interest in the possibility that estrogen improves working memory performance via upregulation of hippocampal activity (Korol and Gold 2007; Hampson and Morley 2013), although not all studies support this (see Janowsky et al. 2000). Further, estrogen might mitigate the negative cognitive effects of glucocorticoids, or at the very least act to positively impact cognition and oppose the negative impacts of glucocorticoids (Herrera and Mather 2015). The cognitive impacts of progesterone are somewhat less clear, but if the stress response is a key component of choking under pressure, there is some evidence that progesterone might positively impact performance under pressure by ameliorating that stress response. Both endogenously produced (Frye and Walf 2002) and exogenously administered (Frye and Walf 2004) progesterone had anxiolytic (stress-relieving) effects in rodents, and a synthetic progesterone derivative was found to specifically modulate the effect of corticotropin-releasing hormone (a key hormone in the HPA cascade) on anxiety behaviors (Britton et al. 1992). This is an important consideration for our understanding of how hormones might produce behavioral responses under pressure: some hormones might affect cognition directly by acting on the cognitive systems needed to complete the task, but some might also improve performance by attenuating the stress response.

Studying hormones and how they might relate to pressure can be difficult in human subjects, as many human studies collect cognitive data and hormone samples from participants once or twice, and use those samples to represent each participant’s hormone levels more generally. However, many hormones, such as cortisol, fluctuate both within a day and across days to reflect events experienced by the individual, which means that a single hormone sample may not be representative of the overall hormonal profile of that individual. Further, human subjects vary widely among a number of dimensions in lifestyle that are difficult to control for in an experimental study (for instance, in diet, physical activity, and daily exposure to stressors), and life histories are often relatively sparse for these individuals. However, such problems can be mitigated in animal models – access to the same animals each day allows for repeated testing and repeated hormonal sampling, and at least in a laboratory setting, diets and other lifestyle factors are usually standardized and recorded in detail throughout the entire life of the animal (although this in and of itself may limit the validity of the results: see Abolins et al. 2017; Rosshart et al. 2017). Therefore, a comparative approach to pressure and its relation to the stress response would allow us to study potential hormonal effects on responses to high-pressure situations in a repeated measures design while also letting us control for lifestyle features that might have an effect on the stress response, and consequently, responses to pressure.

## A comparative approach to pressure

Given the similarities in biological and cognitive systems that are implicated in choking, there is clearly reason to believe that other species experience effects of pressure, and that there is a need for explicit focus on their responses to pressure. While pressure certainly may be implicitly involved in many comparative cognition studies (indeed, reward- and time-pressure are often present when testing other species), almost no research has isolated how that pressure influences cognitive performance and decision-making in a way that effectively isolates it from difficulty. This might be because pressure is an intensely experience-based phenomenon, and some past research in human subjects has relied heavily on self-report measures of pressure. Animals, of course, are unable to self-report internal experiences of pressure, making it challenging to consider pressure in non-human subjects. However, pressure has been correlated with physiological measures as well. Because animals show similar physiological responses to stress and similar cognitive abilities as humans, it follows that high-pressure situations may affect their cognitive systems in similar ways as they do in humans, and that biological correlates of the stress response might covary with performance in these high-pressure situations. However, to test this, we must design cognitive studies that manipulate pressure experimentally, to explore how pressure alone influences performance.

A comparative approach can also help us to distinguish among the explanations for choking that we discussed above, both by providing opportunities to use the repeated biological sampling measures that are often impractical in human samples, and by narrowing down the previously discussed potential explanations for why individuals suffer negative performance consequences as a result of pressure. These explanations for why we choke (distraction, explicit monitoring, and over-arousal) are difficult to tease apart in human subjects because there is both evidence for each of the hypotheses and evidence that multiple of them may play a role. However, studying other species can discriminate among them because many animals show little or no evidence of the cognitive abilities on which some of the theories hinge. If we still observe the choking phenomenon in these species, this allows us to rule out that cognitive skill as being essential (although of course, we cannot rule out that it is involved in humans, even if it is not essential). For instance, while animals show individual distractibility in completing executive function tasks (Matzel and Kolata 2010), as well as a tendency to be overwhelmed by especially desirable visible food rewards when making decisions in some contexts (Cronin 2012), there is no evidence that animals experience anxiety about their own performance as it relates to their self-image. For instance, while some species do show evidence of metacognition through uncertainty monitoring tasks (for instance, bottlenosed dolphins, *Tursiops truncatus*: Smith et al. 1995; honeybees, *Apis mellifera*: Perry and Barron 2013; rhesus macaques, *Macaca mulatta*: Shields et al. 1997, Smith et al. 1997; rats, *Rattus norvegicus*: Foote and Crystal 2007), there is at best minimal evidence in most species that they are aware of themselves as independent beings, or have a self-image (for examples, see Epstein et al. 1981; Anderson and Gallup Jr 2011). Thus, if animal subjects choke under pressure despite this lack of self-awareness, it would rule out the explicit monitoring hypothesis of choking, at least in these species.

One caveat is that reactions to pressure in animals might not be the same as those observed in humans. Because humans do show evidence of more complex cognitive strategies, as well as an added element of cultural and social expectations, it might be that humans have evolved to cope with pressure differently than species that do not show evidence of these abilities. However, the benefit of an animal model for choking lies in distinguishing the cognitive abilities that are necessary to produce failure under pressure from those that might simply influence the likelihood of failure. For instance, if explicit monitoring occurs in humans or other species that *do* show an awareness of the self, yet we see evidence of choking in species where this is not the case, it would suggest that anxious self-reflection is not a necessary prerequisite for pressure-based performance failure, and thus that explicit monitoring is not a necessary feature of choking. This is not to say that explicit monitoring in humans does not play a role in how likely choking is in a human participant, but instead that explicit monitoring is not *necessary* to produce choking in-and-of itself. Therefore, despite this caveat, animal models allow us to assess if human-specific cognitive abilities are a primary factor in the choking phenomenon, or if performance deficits are instead the result of some evolutionarily conserved trait common to modern species that affects cognitive abilities among species.

The most obvious cognitive tasks which we would expect to see an effect of pressure are indeed those which are vulnerable to pressure in the human samples that have been tested—that is, psychomotor abilities (such as we see in human sports) and working memory tasks. Animals frequently need to make coordinated movements in their day-to-day lives, so the ability to make distinct, planned physical movements is a critical one for survival. Therefore, given that much of the research on choking in human subjects takes place in the physical domain of sport, we can start by exploring the effect of pressure on psychomotor ability in non-humans as a relatively direct comparison. One recent study of three rhesus macaques (*Macaca mulatta*) studied choking in a task in which subjects needed to make hand movements to move a cursor toward a target image on a computer monitor within a time constraint that varied by each monkey’s individual ability (Smoulder et al. 2021). All three rhesus macaque subjects showed evidence of choking under pressure when the reward stakes were very high (that is, they were completing “jackpot trials”, in which they received a much larger quantity of reward for successful performance) as compared to when rewards were smaller. Importantly, however, choking only happened when jackpot trials were both very rare within the session (presented on only 5% of trials) and much higher in reward magnitude than the average trial (ten times greater), and neither rarity nor magnitude alone were sufficient to induce a choking effect. Further, on higher reward trials, monkeys spent more time making fine adjustments to achieve the target, presumably to avoid making a mistake. The authors took this result to support an explicit monitoring model of choking, although they did note that there was evidence of both the distraction and overarousal explanations for choking as well (as evidenced by increased initial “false starts” toward the target and increased response times when stakes were high). Importantly, Smoulder and colleagues noted that any source of distraction must have been internal, as monkeys completed the task alone in a darkened room to minimize external distractions.

In fact, this idea of internal distraction fits nicely with a model of pressure that includes the stress response, and we now have evidence from another non-human primate species that the stress response impacts responses to pressure not just in a physical task, but a cognitive one as well. Many animals show evidence of working memory or working-memory-like systems, but there is considerable variability in their ability to complete working memory-based tasks (Matzel and Kolata 2010). Although much of this variability can be attributed to individual differences in working memory capacity, or the innate ability to monitor and work with information as we are holding it in memory, the potential for pressure effects to add to that capacity load is worth exploring in animals, given that humans are known to choke on working memory tasks specifically. In addition, because we expect there to be variation in stress responses as a result of that pressure, we expect the individual variation in biomarkers of stress to interact with variation in working memory capacity. However, before exploring such an interaction, it is important to establish whether there is such variation related to how individuals perform under pressure in any species other than humans.

In a recent paper (Sosnowski et al. 2022), we hypothesized that pressure might explain some of the individual variation that we see across cognitive tasks in animal studies and sought to explore potential sources of that variation in individual differences in the stress response. In this study, tufted capuchin monkeys (*Sapajus [Cebus] apella*) completed a delayed-match-to-sample task in which specific test trials were cued to be high pressure (Sosnowski et al. 2022). To set up these high-pressure trials, the monkeys were first trained to associate a background color cue with a high difficulty, high reward trial, which were intended to be high pressure as compared to their typical trials. Then, during testing sessions, the difference in difficulty between high- and low-pressure trials was removed while keeping the background color cue and higher reward for high-pressure trials only. Therefore, in testing sessions high pressure trials remained higher stakes, but were no longer more difficult, which disentangled difficulty from pressure. There was significant individual variation in how the monkeys performed on the high-pressure trials as compared to their respective performance on low-pressure trials, as well as a significant effect of experience with the pressure condition on that performance. In early sessions, many monkeys were more likely to fail under high-pressure than under low-pressure (choke), but in later sessions many of those monkeys improved their performance under high-pressure, even to the point of performing better under high pressure. Importantly, higher average fecal cortisol throughout the duration of the study was correlated with higher likelihood of choking, especially in early testing sessions (Sosnowski et al. 2022). This suggests that chronic or long-term stress was negatively associated with how individuals performed when under pressure, which is a first step to understanding how cortisol might be involved in developing that response to pressure. However, we still do not know how this long-term stress might interact with in the moment hormonal responses to high-pressure moments, or if this negative relationship can be mitigated in some way.

Another important point from this study was that many monkeys did not choke when their performance was considered overall (Sosnowski et al. 2022). Instead, these individuals seem to thrive, or perform better, on high-pressure trials over the entire duration of testing, or improved their performance in sessions beyond the first few. This improvement in performance for some individuals highlighted the range of response to pressure—some individuals failed to complete the task when the stakes were high, while others were more likely to succeed. While the success of these individuals was almost certainly related to experience with performing under pressure by the final session, it is also probably related to motivation—the high-pressure trials remained highly rewarding even after the difficulty was removed, so monkeys that were able to perform well under pressure may have been particularly motivated to do so. Indeed, in some of these individuals, the data suggested an initial decrement in performance in very early sessions, before a rebound in performance on these high-pressure trials beyond baseline in later ones. This is not unlike results from human subjects—not only do some individuals have the “clutch gene”, as it is colloquially called in sports, but in general, people are less likely to choke when they have experience performing in a high-stakes context (Oudejans and Pijpers 2010).

While there are few non-human animal studies that directly measure the effects of pressure, and even these only in very limited samples, there are also laboratory-based studies that provide indirect evidence for a negative effect of pressure, even when it was not intentionally manipulated. For instance, competitive contexts, in which an individual’s outcome is inherently linked to their opponents, can easily influence individual performance through pressure. Washburn and colleagues (1990) gave two rhesus macaques a computerized task to in which male subjects competed to be the first to “shoot” a digitized on-screen target that appeared at a random location in the middle of the screen. To do so, each monkey manipulated his joystick to move his respective “turret” on the computer screen that continuously shot digital projectiles across the screen at fixed intervals. The goal for each subject was to be the first to manipulate his turret into the correct position to shoot the blue target. Both macaques were working on the same trial, so the task represented a direct competition to shoot the target first. Response times were lower when both macaques were actively engaged in the task (deemed “contested” trials) than a control when only one was attempting it (deemed “uncontested” trials). Critical for a discussion of pressure, however, the macaques also fired more total shots in the “contested” trials, suggesting that they were less likely to shoot accurately on their first shot than in “uncontested” trials (Washburn et al. 1990). While this increase in inaccurate first shots is potentially related to the faster response and not necessarily a by-product of pressure in and of itself, this study shows that competitive and time-constrained pressure influences behavior, and that pressure to perform quickly might be important for understanding performance. To differentiate between these two possibilities, future studies might constrain response times even on uncontested trials to assess if competitive pressure is influencing performance, or if a time constraint alone is enough to induce errors even when we remove the inherent confound of a more difficult task. This would disentangle pressure due to competition from pressure due to the need to act quickly.

Of course, from the perspective of understanding the evolution of responses to pressure, it matters whether pressure effects are observed in the animals’ natural lives, or only in these relatively artificial contexts of the laboratory. Indeed, some field studies suggest that pressure might play a role in decision-making in more natural settings, even though such studies are typically unable to look at pressure directly. For example, when animals are foraging under predation threat, they must make at least a rudimentary cost–benefit analysis to decide when and where to search for food. Clearly, the two competing needs of hunger and avoiding predation must be weighed when deciding if a foraging patch is safe, or alternatively, worth the risk, to search. Some evidence of on-the-spot influences in these decisions comes from pollinator insects, in which there is often a speed-accuracy tradeoff in efficient foraging on flowers (Chittka et al. 2009). For instance, hoverflies (*Sphaerophoria spp.*) change their behavior by repeatedly darting backwards and forwards around a potential foraging location when there is an unknown risk of crab spider (*Thomisus labefactus*) predation, as compared to when the crab spider is clearly visible on the flower (Yokoi and Fujisaki 2009). This darting behavior results in a severe drop in efficiency—so individuals must decide when to stop hesitating when there is no evidence of a predator. Making a *wrong* or hasty decision, however, clearly has swift negative consequences for the hoverfly—therefore, the hoverfly’s survival outcome depends on its ability to make correct decisions, introducing an element of pressure. Such hesitation under predator threat also is supported by laboratory studies in artificial meadows in bumblebees (*Bombus terrestris*), which not only exhibit a speed-accuracy tradeoff (Chittka et al. 2003) but also exhibit “false alarms” when under pressure (Ings and Chittka 2008), as well as guppies, in which hastiness to make a choice is affected by predation experience (Burns and Rodd 2008). While hoverflies and bumblebees may not have a similar enough cognitive architecture to experience a similar choking effect (although new research on insects suggests complex cognitive behaviors, e.g., numerical understanding in honeybees, *Apis mellifera*: Dacke and Srinivasan 2008; Howard et al. 2018; or individual recognition of conspecifics in the paper wasp, *Polistes fuscatus*: Tibbetts 2002), similar sorts of tradeoffs exist in nearly every species.

Further, in all of these species, predation threat likely introduces pressure into foraging situations, resulting in this observed speed-accuracy tradeoff. There is also evidence for a similar cost–benefit analyses in other contexts. For instance, free-living great tits (*Parus major*) are influenced by threat of starvation when choosing to return to feeders that have been previously predator disturbed (Mathot et al. 2015). Hesitating to return to a feeder too long can result in a disastrous loss of energy or starvation, so the ability to make an appropriate decision whether to risk a trip to the feeder is key to a bird’s survival, clearly introducing an element of pressure as we have defined it. However, it is difficult to directly induce pressure in field experiments, due to confounds of difficulty or ethical concerns, so we have yet to examine pressure directly in natural settings. However, given the high stakes of these decisions, and the fact that speed-accuracy tradeoffs have been taken to suggest the presence of pressure in laboratory settings, careful development of methods that test if pressure to make good decisions plays a role in these ecologically relevant situations will be key to exploring pressure effects in non-human species.

## Responses to pressure as an individual factor in comparative cognition

Comparative cognition research often studies a particular sample (or in some cases, several samples), which can make it difficult to make broad generalizations at a species level, due to the range of responses (Thornton and Lukas 2012), or determine what is due to individual differences. Indeed, as in humans, context and history can strongly influence responses, thus specific animal populations may have different responses to the same stimuli or contexts (for instance, STRANGE samples: Farrar and Ostojić 2021; Webster and Rutz 2020), making it important to consider results not just at a species-level, but at a population or individual level. Of course, the goal is to begin to expand our research samples to a variety of samples that live across ecological contexts, so that we can start to make generalizations about the species as well as those individual differences and contextual variables that influence individual’s outcomes. One challenge, however, is determining which of these factors influencing individual variability are theoretically interesting and usefully predict variation. What we know about choking from the human samples that have been studied suggests that individual reactions to pressure might be one such factor.

For instance, many decision-making tasks are based on the assumption that individuals will maximize their outcomes by choosing the “best” response, something that animals and humans fail to do consistently (Waksberg et al. 2009; Zentall 2016). There are many possible reasons for this. It could be that animals are making a mistake, or do not understand the task, although that seems unlikely given many species’ expertise at these tasks. It also may be that they are showing some of the same decision-making biases seen in humans (De Petrillo and Rosati 2019; Williamson et al. 2019) or that rules of thumb are good enough most of the time (Watzek and Brosnan 2018), and of course animals show individual differences based on demographic factors, personality (Hopper et al. 2014) and differences in various cognitive abilities. Another possibility, inherent in these decision-making tasks, is that each individual’s outcome depends on their own decision-making performance—in other words, there is pressure within these tasks for animals to maximize their outcome. As we know that some humans (and monkeys) are more prone to choking under pressure than others (and indeed, that increasing that pressure can lead to suboptimal choices: (Jones et al. 2011), it follows that some animal subjects would also be better at coping with pressure than others when completing decision-making tasks. Thus, at least some of the differences in how individuals respond to pressure could be related to individual variation in the HPA stress response or their interaction with other sources of individual variation, especially if biological responses to pressure cause a breakdown in cognitive abilities such as working memory that are needed to make the correct choice.

Pressure may also play a specific role in a variety of other cognitive tasks for which we see substantial individual differences. For instance, sequential learning requires not just working memory, but also planning and metacognition. Sequential learning and responding has been documented in a number of non-human species using visual sequencing tasks, in which an individual needs to select stimuli in a pre-trained order. Within this type of task, a number of non-human primate species can learn sequences of varying lengths, including chimpanzees (Ohshiba 1997; Biro and Matsuzawa 1999; Beran et al. 2004; Inoue and Matsuzawa 2009), Japanese macaques (Ohshiba 1997), rhesus macaques (Beran et al. 2004), and capuchin monkeys (Beran and Parrish 2012). In addition, many non-primates show a similar sequence-learning ability (for instance, pigeons, *Columba livia domestica*: Richardson and Warzak 1981; or rats, discussed in Keeler et al. 2014). While associative learning strategies can be used if a stimulus disappears once selected, if all stimuli remain on the screen for the duration of the trial (even after they have been correctly selected in the sequence) or all stimuli are hidden after the selection of the first stimulus, then an individual must engage working memory to monitor and keep a running tab of which stimuli have already been selected in a given trial to correctly reach the end of the sequence. Within each species and sample, there is significant individual variability in subjects’ actual performance while learning and completing the tasks. As an example, capuchin monkeys varied in their ability to successfully complete a sequence of three to four stimuli when the stimuli were hidden after the correct selection of the first stimulus (Beran and Parrish 2012), as compared to a more simple trial type in which all stimuli remained visible. While of course such differences may be due to individual variation in working memory capacity or planning, these visually distinct and more difficult trials may also have induced pressure, leading some individuals to performing above chance and others below. Future studies could test this using a response time constraint on some trials, which, in human subjects, impacts physical sequence completion (Koedijker et al. 2011).

## Future directions

The similarities in cognitive systems that are susceptible to pressure effects and the conserved biological mechanisms suggest that pressure may have a stronger impact on results in comparative cognition than is currently recognized. Indeed, we now have some direct evidence that some non-human species are susceptible to pressure. However, this evidence comes from only two samples of animals from two non-human primate species—therefore there are some obvious next steps. First, the samples from both studies are very small and come from a specific group of captive monkeys. Thus, we need a broader sample of these species to generalize more broadly to the species (the same, of course, is true for human studies!). Second, an obvious next step is to expand to other species, and in particular, beyond the primates. It makes sense to begin with those for which evidence already exists that they possess a working-memory-like system. If these primates’ responses to pressure are influencing performance in working memory tasks, we predict a correlation between working memory performance and markers of the stress response, such as hormonal changes or behavioral changes, as we have observed in capuchin monkeys. It would also be good to explore whether other systems are prone to pressure influences. For instance, we also have evidence that psychomotor function might be impacted in rhesus macaques (Smoulder et al. 2021), but what about other cognitive systems that are, like working memory, related to executive functioning (i.e., inhibition of impulse)? We have growing evidence that working memory capacity may predict general cognitive ability across a range of domains in animals (Kolata et al. 2005), so pressure effects may have explanatory value in tasks spanning multiple domains in which we see individual variation in performance or decision-making.

It is almost certain that context is an important variable in eliciting pressure effects. The work thus far in non-human species has focused on a non-social context in which the subject is playing for him- or her-self alone, and the consequences of failure affect only him or her. It would be interesting to see if group-living species are more sensitive to pressure when failure affects a conspecific groupmate as well as the subject, or if the conspecific adds additional motivation to override an initial deficit in cognitive ability. In humans, there seems to be a negative audience effect; social attention apparently increases the pressure of a situation, even if that attention is supportive (Butler and Baumeister 1998; Wallace et al. 2005). In animals, who show less evidence of possessing consciousness about their performance (i.e., no self-esteem), it is less clear how a social context might influence pressure responses. It might be that, like in humans, a social context hinders performance, or it could be that the presence of an audience simply changes the context surrounding a decision (for instance, showing a video of a high- or low-ranking conspecific’s face changes chimpanzees’ risk preferences: Proctor et al 2017). Thus, exploring how social contexts influence pressure responses in non-humans could further tease apart what exactly is happening cognitively when people choke or thrive under pressure.

Further, if some hormones have a negative relationship with performance under pressure (like cortisol), other hormones might mitigate the negative relationship between cortisol and performance. Given the social buffering hypothesis (Cohen and Wills 1985), we might look to hormones involved in these social contexts, such as oxytocin, a neuropeptide hormone that has been widely studied in the context of maternal-infant bonding as well as more general social behavior. One explanation of how oxytocin is implicated in social cognition is the anxiety reduction hypothesis, which suggests that oxytocin promotes sociality by having an anxiolytic effect when released in response to affiliative behavior (Heinrichs and Domes 2008). For instance, oxytocin is released in response to psychologically stressful situations in rats (Neumann et al. 2000), and its release is associated with decreased reactivity to those stressful situations in the HPA axis (Neumann 2002; Heinrichs and Domes 2008). Importantly for our discussion of pressure, administration of exogenous oxytocin in conjunction with positive social contact (which may induce release of endogenous oxytocin in and of itself) was associated with suppressed cortisol response and a behavioral decrease in reactivity to psychosocial stress in human subjects (Heinrichs et al. 2003). In fact, the connection between oxytocin release and improvement in pressure-induced anxiety has already been demonstrated in human subjects, who were administered exogenous oxytocin before undergoing a public speaking test. Subjects self-reported reduction of anxiety and showed physiological signs of decreased arousal prior to and during the public speaking test (de Oliveira et al. 2012). However, this study, like many studies with human subjects, was able to collect data and physiological samples from a single experimental session only. Further, the manipulation involved only exogenous oxytocin administration, rather than including a manipulation in which endogenous oxytocin was increased as the result of a social interaction. Using an animal model to study this relationship would allow for more consistent hormone sampling, as in captivity, animals are often able to be tested multiple times. In addition, the opportunity to induce social behaviors to naturally increase endogenous oxytocin is present in at least one species. Tufted capuchin monkeys will reliably engage in social contact while fur-rubbing, which has been shown to reliably increase endogenous urinary oxytocin (Benítez et al. 2018; Sosnowski et al. in revision). Therefore, the opportunity to assess if social interaction (and the resulting oxytocin increase) impact reactions to acute stressors like pressure is a fertile one. If the anxiety reduction hypothesis holds in other species, then we would expect to see an improvement in cognitive performance under high pressure after a socially rewarding activity, such as engaging in affiliative behavior with a bonded conspecific, as compared to when conspecifics are not present.

One final avenue of future research is collaborative efforts among laboratory and field researchers to explore pressure as an explanation for suboptimal behavior. The decisions that animals make in their natural environment probably represent their most “typical” cognitive abilities (or how those typical abilities manifest behaviorally in ecologically relevant situations), but typical abilities might not represent the full extent of what animals are capable of. Laboratory settings offer invaluable opportunities to test animal cognition in a controlled way, to probe specific aspects of the decision-making process, and to use animals as a model system for understanding the effects of pressure as a part of that controlled approach. In turn, field studies can apply this understanding to explore high-stress situations that may be more comparable to evolutionarily relevant events, informing which situations are most likely to induce responses to pressure in other species. By including pressure in models of cognition in producing wild behavior, we can better understand why some individuals might be more successful when success hinges on deciding correctly, for instance when deciding whether to flee or to stand and fight, or why some individuals are able to manage complex social encounters more effectively than others. In addition, the addition of naturalistic observation in high-pressure situations would provide an ecologically relevant understanding of pressure that has the important added benefit of expanding the body of work to more samples within a species in its natural environment.

The exploration of pressure in non-human species has just begun to take shape, with the first studies from non-humans providing evidence that they are, like humans, susceptible to pressure effects. Further, these effects are present when performing both physical actions and cognitive tasks, again suggesting a strong similarity with human responses. However, much work remains to be done to understand the extent of other species’ responses, both in terms of the species who show the effect and the contexts in which it occurs, and fully integrate an understanding of pressure into our understanding of animal decision-making and behavior. In particular, it will be important to explore the effects of pressure beyond the primate order to better understand which cognitive systems are involved. No matter what we find, a better understanding of pressure in non-human species will help us to better understand choking (and thriving) under pressure in humans and how pressure may have played a role in the evolution of cognition.
